# Admission Systolic Blood Pressure and In-hospital Mortality in Acute Type A Aortic Dissection: A Retrospective Observational Study

**DOI:** 10.3389/fmed.2021.542212

**Published:** 2021-07-20

**Authors:** Guifang Yang, Wen Peng, Yang Zhou, Huaping He, Xiaogao Pan, Xizhao Li, Xiangping Chai

**Affiliations:** ^1^Department of Emergency Medicine, The Second Xiangya Hospital, Central South University, Changsha, China; ^2^Emergency Medicine and Difficult Diseases Institute, Central South University, Changsha, China; ^3^Department of Cardiovascular Surgery, The Second Xiangya Hospital, Central South University, Changsha, China

**Keywords:** systolic blood pressure, SBP, aortic dissection, AAD, in-hospital mortality

## Abstract

**Background:** Evidence between admission systolic blood pressure (SBP) and in-hospital deaths in acute type A aortic dissection (AAD) patients is inadequate. Here, we examined the relationship between SBP and in-hospital deaths in AAD patients.

**Methods:** 703 AAD patients were enrolled from January 2014 to December 2018. The independent and dependent variables targeted were admission SBP and in-hospital deaths, respectively. Gender, age, body mass index (BMI), chronic renal insufficiency, smoking, hypertension, diabetes, laboratory indicators, and management were used as covariates.

**Results:** The 703 participants had a mean age of 50.48 ± 11.35. About 76.24% of the participants were male. After adjusting for confounders, there was a negative correlation between AAD patients' admission SBP and in-hospital deaths (OR = 0.88, 95%CI 0.80–0.96). Consequently, a non-linear relationship of point 120 (mmHg) was detected between admission SBP and in-hospital deaths for AAD patients. Confidence intervals and effect sizes of the right (SBP >120 mmHg) and left (SBP ≤ 120 mmHg) sides of the inflection point were 0.96 (0.85–1.09) and 0.67 (0.51–0.88), respectively. The change in the male population and non-diabetes people was more pronounced according to subgroup analysis.

**Conclusions:** Correlation between admission SBP and in-hospital mortality of AAD patients is non-linear. SBP negatively correlated with in-hospital mortality when ≤120 mmHg.

## Introduction

Numerous studies have identified blood pressure as a key determinant of adverse events in cardiovascular disease patients (1, 2). Various studies have observed the J-curve phenomenon amid blood pressure serial levels and adverse cardiovascular events like in-hospital mortality (3, 4). Many observational studies show that admission systolic blood pressure (SBP) is associated with the risk of death in acute cardiovascular conditions like acute heart failure (5) and cardiogenic shock (6, 7). Additionally, admission SBP is integrated into most danger scoring models in patients with acute coronary syndromes (8). Conversely, in-hospital deaths in patients with acute type A aortic dissection (AAD) are unclear in relation to SBP at admission. Here, we examined the relationship between SBP admission levels and in-hospital mortality among unselected consecutive AAD patients after adjusting for potential confounding factors.

## Methods And Participants

### Study Design

This retrospective, observational study used baseline admission SBP as independent variable and all-cause in-hospital death as dependent variable.

### Study Population

We non-selectively and consecutively collected data for all participants at the Second Xiangya Hospital of Central South University, Changsha, Hunan, China. Anonymous data were compiled from the electronic hospital medical record system. Ethical approval for the study was provided by the hospital's institutional review board. Informed consent was waived because the study was retrospective.

The study involves 703 in-patients treated at the hospital from January 2014 to December 2018. Diagnosis was mainly based on 2014 ESC guidelines on the treatment and diagnosis of aortic ailments. Any dissection that involved the ascending aorta with presentation within 14 days of symptom onset was defined as AAD. The diagnosis of AAD was confirmed by imaging like Computed tomography (CT) or Magnetic resonance imaging (MRI). Inclusion criteria were hospital admission for acute type A aortic dissection patients within ≤ 14 days after symptoms onset. The following were used as exclusion criteria: (1) unfinished blood pressure tests, (2) presence of intramural hematoma, which a haematoma develops in the media of the aortic wall in the absence of an false lumen and intimal tear. Intramural haematoma is diagnosed in the presence of a circular or crescent-shaped thickening of more than 5 mm of the aortic wall in the absence of detectable blood flow, (3) presence of symptoms for >14 days.

### Variables

Admission SBP for all AAD participants was measured at baseline. In-hospital mortality was described as all-cause death during admission. Study covariates involved general information, demographic data, blood biochemistry, medical imaging examination and treatment variables that can affect admission SBP, or in-hospital mortality. Variables for the construction of the fully adjusted model were: (1) continuous variables (age, BMI, time to presentation, aortic diameter, triglyceride (TG), high-density lipoprotein (HDL), ejection fraction (EF), low-density lipoprotein (LDL), creatinine (Cr), total cholesterol (TC), and obtained at baseline), and (2) categorical variables (including smoking, gender, diabetes, Marfan syndrome, bicuspid aortic valve, hypertension, stroke, atherosclerosis, aortic regurgitation, chronic renal insufficiency (CRI), abdominal vessel involvement, arch vessel involvement, symptom, and management.

### Missing Data Addressing

We performed multiple multivariable imputations to address missing data in order to maximize statistical power and minimize bias. The data analysis had no covariates with missing data. In addition, five imputed datasets with chained equations were created using MICE software package (9). Sensitivity analysis found no significant differences between the generated complete data and raw data. Thus, all multivariable analyses results based on the imputed datasets were combined with Rubin's rules.

### Statistical Analysis

Continuous variables are shown as mean ± SD (normal distribution) or medium (25th, 75th) [skewed distribution]. Categorical variables are shown as percentage. Kruskal Wallis H test (skewed distribution), chi-squared test (categorical variables) or ANOVA (One way) were used to analyze normally distributed data, particularly variations between different admission systolic blood pressure groups (Quartile). To investigate whether admission systolic blood pressure correlated with in-hospital mortality in certain members, statistical analyses were done in three key steps. Step 1: Multivariate and univariate regression (linear) models engaged. Three additional models were constructed. A crude model without covariates adjustment was made. Model I was adjusted only for sociodemographic data. The second model was made by adding the covariates as shown in Table 1 to the first model. Step 2 addressed non-linearity in admission SBP and in-hospital mortality. Fitting of an additive-generalized model and penalized spline method (smooth curve) was done. In case any detection of non-linearity was observed, the point of inflection was calculated using a recursive algorithm and a linear two-piece regression constructed. This was done on the inflection point for both sides. For the likelihood log-ratio test, the best fit model was checked on the values of P. Step 3: a stratified linear regression model was used for subgroup analyses. First, continuous variable were changed to categorical variables as stated in the clinical quartile (cut point) and an interaction test was done. A test on the likelihood ratio followed the checks done on the modification of effect for those of indicators on the subgroup. A sensitivity study was used to confirm the stoutness of data analysis that converted admission SBP to a categorical variable and the trend's *p value* calculated. The aim was to detect the likelihood of non-linearity and to affirm SBP admission results as a continuous variable. Survival curves were constructed using Kaplan–Meier analysis and parallels with the test on log-rank. EmpowerStats (http://www.empowerstats.com, X&Y Inc Solutions, Boston, MA) and R (http://www.r-project.org) were used for statistical analyses. *P* = <0.05 (two-sided) was considered statistically significant.

**Table 1:** Basline characteristics of the patients (*N* = 703).

**Characteristic**	**Systolic blood pressure (mmHg) (Quarter)**				***P*-value**
	**Q1 (64–125)**	**Q2 (126–144)**	**Q3 (145–164)**	**Q4 (165–233)**	
No. of patients	210	163	156	174	
Age (years, mean ± sd)	50.31 ± 12.10	52.63 ± 10.71	49.42 ± 11.88	49.63 ± 10.29	0.041
Gender (female)	52 (24.76%)	41 (25.15%)	32 (20.51%)	42 (24.14%)	0.750
BMI (Kg/m^2^, mean ± sd)	23.76 ± 3.58	25.16 ± 4.44	25.72 ± 4.66	26.56 ± 4.44	<0.001
Smoking	57 (27.14%)	42 (25.77%)	53 (33.97%)	45 (25.86%)	0.304
Hypertension	112 (53.33%)	99 (60.74%)	119 (76.28%)	148 (85.06%)	<0.001
Diabetes	8 (3.81%)	5 (3.07%)	4 (2.56%)	5 (2.87%)	0.913
Marfan syndrome	10 (4.76%)	3 (1.84%)	6 (3.85%)	1 (0.57%)	0.066
Bicuspid aortic valve	3 (1.43%)	3 (1.84%)	1 (0.64%)	1 (0.57%)	0.636
CRI	2 (0.95%)	5 (3.07%)	1 (0.64%)	10 (5.75%)	0.008
Stroke	7 (3.33%)	5 (3.07%)	4 (2.56%)	8 (4.60%)	0.766
Atherosclerosis	16 (7.62%)	17 (10.43%)	8 (5.13%)	7 (4.02%)	0.094
Time to presentation (h, median [Q1–Q3])	15.00 (9.00–36.75)	24.00 (13.00–48.50)	20.00 (10.50–48.00)	18.00 (10.00–36.00)	0.160
SBP (mmHg, mean ± sd)	106.70 ± 14.30	135.14 ± 5.52	154.63 ± 5.45	182.75 ± 15.72	<0.001
DBP (mmHg, mean ± sd)	62.05 ± 13.04	74.22 ± 13.02	81.24 ± 13.62	97.06 ± 17.32	<0.001
Aortic diameter (mm)	46.50 ± 11.06	47.41 ± 11.61	45.23 ± 10.07	42.73 ± 7.59	<0.001
Aortic regurgitation	99 (47.14%)	80 (49.08%)	63 (40.38%)	72 (41.38%)	0.293
Abdominal vessel involvement	52 (24.76%)	40 (24.54%)	48 (30.77%)	43 (24.71%)	0.505
Arch vessel involvement	64 (30.48%)	49 (30.06%)	55 (35.26%)	53 (30.46%)	0.714
EF value (%)	63.98 ± 10.08	65.13 ± 7.83	64.81 ± 8.26	66.43 ± 5.38	0.066
Symptom					0.062
Chest pain	171 (81.43%)	135 (82.82%)	131 (83.97%)	144 (82.76%)	
Back pain	5 (2.38%)	7 (4.29%)	2 (1.28%)	8 (4.60%)	
Abdominal pain	7 (3.33%)	4 (2.45%)	3 (1.92%)	11 (6.32%)	
Syncope	10 (4.76%)	3 (1.84%)	2 (1.28%)	3 (1.72%)	
Cr (umol/L)	116.37 ± 85.08	113.92 ± 112.81	98.81 ± 83.35	145.95 ± 201.93	0.009
TG (mmol/L)	1.63 ± 0.95	1.52 ± 0.91	1.56 ± 0.90	1.59 ± 0.95	0.679
TC (mmol/L)	3.72 ± 0.92	3.81 ± 0.78	3.90 ± 0.94	4.16 ± 0.94	<0.001
HDL (mmol/L)	1.07 ± 0.30	1.13 ± 0.31	1.21 ± 0.42	1.23 ± 0.35	<0.001
LDL (mmol/L)	1.89 ± 0.83	1.89 ± 0.73	2.02 ± 0.87	2.18 ± 0.83	0.002
Management					0.494
Medical	75 (35.71%)	48 (29.45%)	48 (30.77%)	48 (27.59%)	
Endovascular	11 (5.24%)	14 (8.59%)	8 (5.13%)	10 (5.75%)	
Surgical	124 (59.05%)	101 (61.96%)	100 (64.10%)	116 (66.67%)	
Mortality (overall)					0.048
Survivor	124 (59.05%)	112 (68.71%)	108 (69.23%)	124 (71.26%)	
Non-survivor	86 (40.95%)	51 (31.29%)	48 (30.77%)	50 (28.74%)	
Mortality (operation)					0.076
Survivor	115 (85.19%)	106 (92.17%)	101 (93.52%)	117 (92.86%)	
Non-survivor	20 (14.81%)	9 (7.83%)	7 (6.48%)	9 (7.14%)	

*BMI, body mass index; SBP, systolic blood pressure; DBP, diastole blood pressure; Cr, creatinine; TG, triglyceride; TC, total cholesterol; HDL, high-density lipoprotein; LDL, low-density lipoprotein; CRI, chronic renal insufficiency; EF, ejection fraction*.

## Results

### Characteristics of Selected Patients at Baseline

**Figure 1:**
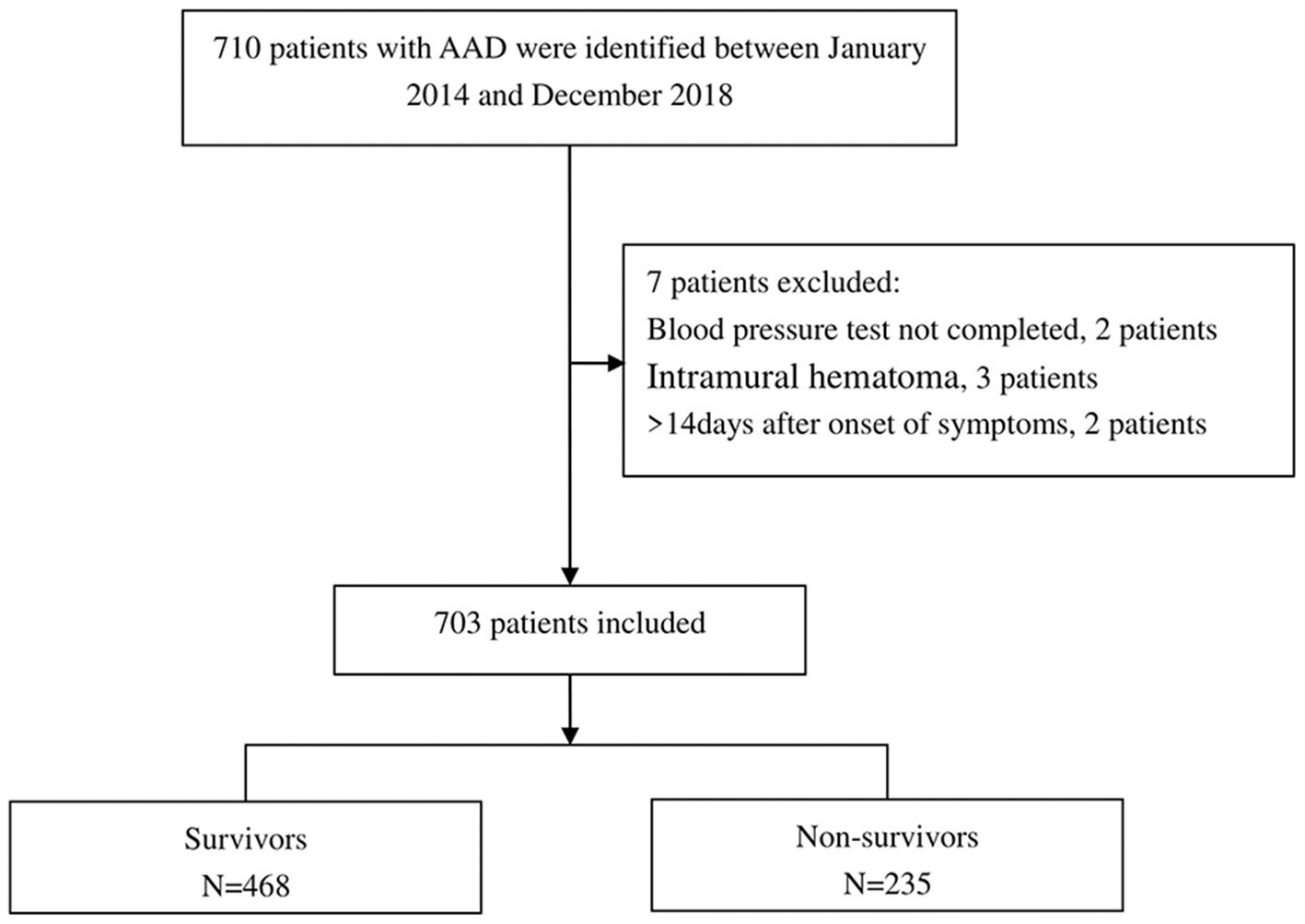
Flow chart of patient enrollment.

Based on exclusion and inclusion criteria, 703 participants were included (Figure 1). The baseline characteristics of these selected participants are shown in Table 1 in reference to the quartile admission SBP. The participant's average age was 50.48 ± 11.35 years and 76.24% of them were male. No statistically significant differences were observed for smoking, gender, Marfan syndrome, diabetes, bicuspid aortic valve, stroke, atherosclerosis, time to presentation, aortic regurgitation, abdominal vessel involvement, arch vessel involvement, EF value, symptom, TG, and management among different admission SBP clusters (all *p values* = >0.05). Contributors with the uppermost group of admission SBP (Q4) had higher values in BMI, DBP, Cr, TC, HDL, LDL, hypertension, and CRI relative to the other groups. Similar patterns were observed for age and aortic diameter in Q2 groups, and mortality in Q1 groups. Supplementary Tables 1, 2 show the causes of death in each quartile of operation (endovascular and surgical) patients and the rates of in-hospital complications for each quartile of medical management patients, respectively.

### Univariate Analysis

Univariate analyses results (Table 2) revealed that gender (0.97, 0.67–1.40), BMI (1.00, 0.97–1.04), smoking (0.83, 0.58–1.18), hypertension (1.32, 0.94–1.86), diabetes (1.39, 0.59–3.31), mafan syndrome (1.34, 0.54–3.32), bicuspid aortic valve (0.66, 0.13–3.30), CRI (2.56, 0.99–6.56), stroke (2.04, 0.90–4.62), time to presentation (1.00, 0.99–1.00), aortic diameter (1.00, 0.99–1.02), aortic regurgitation (0.88, 0.64–1.21), abdominal vessel involvement (0.78, 0.54–1.13), arch vessel involvement (0.89, 0.63–1.25), EF value (0.99, 0.97–1.01), back pain (0.72, 0.28–1.87), abdominal pain (0.61, 0.24–1.54), syncope (1.22, 0.47–3.20), TG (1.16, 0.98–1.36), TC (1.04, 0.87–1.23), and LDL (0.83, 0.68–1.01) were not concomitant with the outcome variable. Additionally, SBP (0.91, 0.86–0.96), DBP (0.98, 0.97–0.99), HDL (0.50, 0.30–0.84), surgical (0.02, 0.01–0.03) and endovascular (0.01, 0.00–0.04) were negatively correlated with the outcome variable. Contrary to this, univariate analysis showed that age (1.02, 1.01–1.04), atherosclerosis (2.10, 1.17–3.79) and Cr (1.00, 1.00–1.00) correlated with the outcome variable.

**Table 2:** Univariate analysis for in-hospital mortality.

	**Statistics**	**OR (95%CI)**	***P*-value**
Age (years)	50.48 ± 11.35	1.02 (1.01, 1.04)	<0.001
Gender (female)	167 (23.76%)	0.97 (0.67, 1.40)	0.877
BMI (Kg/m^2^)	25.21 ± 4.38	1.00 (0.97, 1.04)	0.817
Smoking	197 (28.02%)	0.83 (0.58, 1.18)	0.298
Hypertension	478 (67.99%)	1.32 (0.94, 1.86)	0.115
Diabetes	22 (3.13%)	1.39 (0.59, 3.31)	0.452
Marfan syndrome	20 (2.84%)	1.34 (0.54, 3.32)	0.529
Bicuspid aortic valve	8 (1.14%)	0.66 (0.13, 3.30)	0.614
CRI	18 (2.56%)	2.56 (0.99, 6.56)	0.051
Stroke	24 (3.41%)	2.04 (0.90, 4.62)	0.086
Atherosclerosis	48 (6.83%)	2.10 (1.17, 3.79)	0.013
Time to presentation (h, median [Q1**–**Q3])	20.00 (10.00**–**48.00)	1.00 (0.99, 1.00)	0.149
SBP(per 10 mmHg)	14.28 ± 3.11	0.91 (0.86, 0.96)	<0.001
DBP (mmHg)	77.80 ± 19.40	0.98 (0.97, 0.99)	<0.001
Aortic diameter (mm)	45.46 ± 10.32	1.00 (0.99, 1.02)	0.621
Aortic regurgitation	314 (44.67%)	0.88 (0.64, 1.21)	0.425
Abdominal vessel involvement	183 (26.03%)	0.78 (0.54, 1.13)	0.192
Arch vessel involvement	221 (31.44%)	0.89 (0.63, 1.25)	0.505
EF value (%)	65.07 ± 8.14	0.99 (0.97, 1.01)	0.345
Symptom			
Chest pain	581 (82.65%)	Ref	
Back pain	22 (3.13%)	0.72 (0.28, 1.87)	0.499
Abdominal pain	25 (3.56%)	0.61 (0.24, 1.54)	0.293
Syncope	18 (2.56%)	1.22 (0.47, 3.20)	0.684
Cr (umol/L)	119.23 ± 130.21	1.00 (1.00, 1.00)	0.003
TG (mmol/L)	1.58 ± 0.93	1.16 (0.98, 1.36)	0.087
TC (mmol/L)	3.89 ± 0.91	1.04 (0.87, 1.23)	0.678
HDL (mmol/L)	1.16 ± 0.35	0.50 (0.30, 0.84)	0.008
LDL (mmol/L)	1.99 ± 0.83	0.83 (0.68, 1.01)	0.057
Management			
Medical	219 (31.15%)	Ref	
Endovascular	43 (6.12%)	0.01 (0.00, 0.04)	<0.001
Surgical	441 (62.73%)	0.02 (0.01, 0.03)	<0.001

*BMI, body mass index; SBP, systolic blood pressure; DBP, diastole blood pressure; Cr, creatinine; TG, triglyceride; TC, total cholesterol; HDL, high-density lipoprotein; LDL, low-density lipoprotein; CRI, chronic renal insufficiency; EF, ejection fraction*.

### Results of the Unadjusted and Adjusted Model

Three models were created in this experiment to examine the autonomous effects of admission SBP on in-hospital mortality after modifying for possible confounders. The effect values (OR) and 95% confidence intervals for these three equations are shown in Table 3. In the non-adjusted model (crude model), for every 10 mmHg rise in admission SBP, in-hospital death reduced by 9% (0.91, 95% CI 0.86–0.96). In the minimum-adjusted model (model I), admission SBP was greater by 10 mmHg and in-hospital mortality reduced by 9% (0.91, 95% CI 0.86–0.96). In the fully adjusted model (model II) (adjusted covariates are shown in Table 1, except DBP), for each additional 10 mmHg of admission SBP, in-hospital mortality reduced by 12% (0.88, 95% CI 0.80–0.96). We also converted admission SBP from a continuous variable to a categorical variable (Quartile). The P for trend of admission SBP with categorical variables in the fully adjusted model was constant with the result with admission SBP as a constant variable. However, when the admission SBP enters the fully-adjusted model as a categorical variable, the trend of the effective value in the different admission SBP group is non-equidistant. Based on this non-equidistant changes in effect size, there may be a non-linear relationship between in-hospital mortality and SBP admission.

**Table 3:** Relationship between systolic blood pressure and in-hospital mortality in different models.

**Exposure**	**Crude Model (OR, 95%CI, *P*)**	**Model I (OR, 95%CI, *P*)**	**Model II (OR, 95%CI, *P*)**
SBP (mmHg, per 10 increments)	0.91 (0.86, 0.96) <0.001	0.91 (0.86, 0.96) <0.001	0.88 (0.80, 0.96) 0.005
SBP (mmHg) (quarter)			
Q1	Ref	Ref	Ref
Q2	0.66 (0.43, 1.01) 0.055	0.61 (0.40, 0.95) 0.029	0.60 (0.30, 1.21) 0.156
Q3	0.64 (0.41, 0.99) 0.046	0.65 (0.42, 1.01) 0.053	0.47 (0.22, 1.00) 0.050
Q3	0.58 (0.38, 0.89) 0.013	0.59 (0.38, 0.91) 0.016	0.44 (0.21, 0.94) 0.035
*P* for trend	0.012	0.018	0.025

*Crude Model adjusted for none. Model I adjusted for age and gender. Model II adjusted for age, gender, BMI, smoking, hypertension, diabetes, Marfan syndrome, Bicuspid aortic valve, CRI, stroke, atherosclerosis, time to presentation, aortic diameter, aortic regurgitation, abdominal vessel involvement, arch vessel involvement, EF value, symptom, Cr, TG, TC, HDL, LDL, and management. BMI, body mass index; SBP, systolic blood pressure; Cr, creatinine; TG, triglyceride; TC, total cholesterol; HDL, high-density lipoprotein; LDL, low-density lipoprotein; CRI, chronic renal insufficiency; EF, ejection fraction*.

### The Non-linearity Results Between In-hospital Mortality and Admission SBP

**Figure 2:**
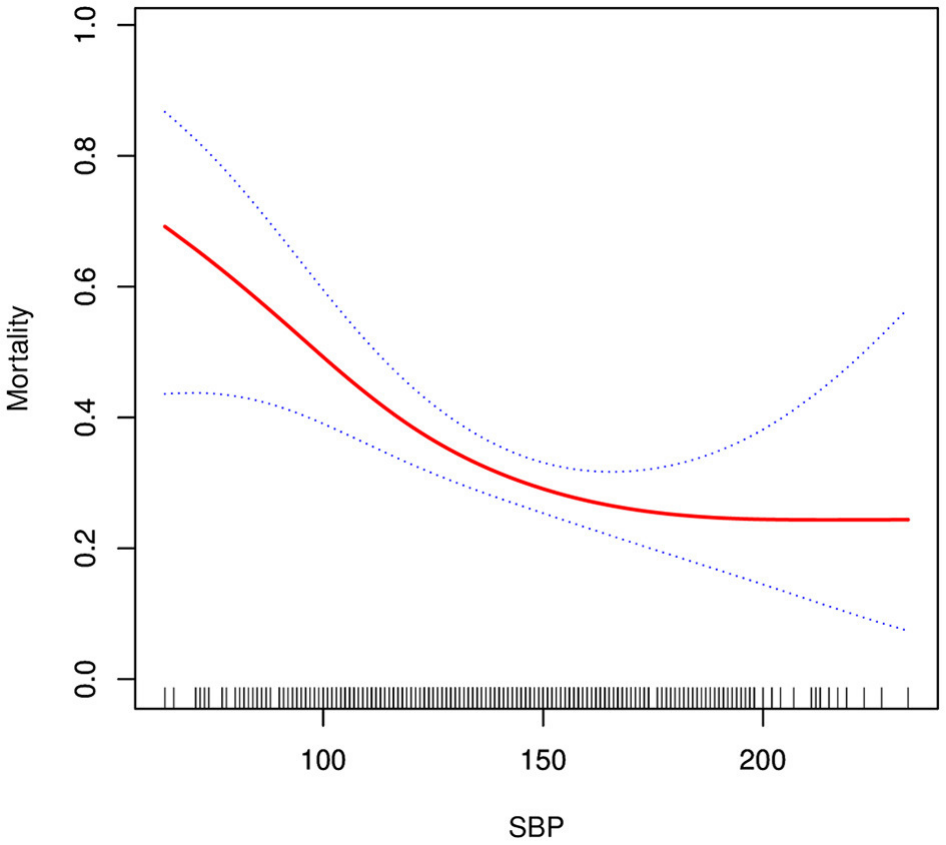
Association between systolic blood pressure and in-hospital mortality. A non-linear association between systolic blood pressure and in-hospital mortality was found (*P* = 0.009) in a generalized additive model (GAM). The solid red line represents the smooth curve fit between variables. Blue bands represent the 95% confidence interval from the fit. All adjusted for age, gender, BMI, smoking, hypertension, diabetes, Marfan syndrome, Bicuspid aortic valve, CRI, stroke, atherosclerosis, time to presentation, aortic diameter, aortic regurgitation, abdominal vessel involvement, arch vessel involvement, EF value, symptom, Cr, TG, TC, HDL, LDL, and management.

The current study examined the non-linear correlation between in-hospital mortality and admission SBP (Table 4, Figure 2). The smooth curve outcome revealed that the relationship between in-hospital mortality and admission SBP was non-linear (after amending for covariates presented in **Table l** excepted DBP). We fit the association between in-hospital mortality and admission SBP using linear regression model and two-piecewise linear regression model, respectively. The *p*-*value* for the log-likelihood ratio test was <0.05. This result indicates dual piecewise linear regression was more appropriate for fitting the association between admission SBP and in-hospital mortality since it perfectly represents association between admission SBP and in-hospital mortality. The premeditated inflection point was 120 (mmHg) through two-piecewise linear regression and recursive algorithm. On the left side of the inflection point (SBP ≤ 120 mmHg), effect size and 95% CI was 0.67 and 0.51–0.88, respectively (SBP per 10 increments). On the right side of the inflection point (SBP >120 mmHg), effect size and 95% CI was 0.96 0.85–1.09, correspondingly (SBP per 10 increments).

**Table 4:** The results of the two-piecewise linear model (SBP per 10 increments).

	**Mortality (OR, 95%CI)**	***P*-value**
Fitting model by standard linear regression	0.88 (0.80, 0.96)	0.005
Fitting model by two-piecewise linear regression		
Inflection point of SBP	120	
≤ 120	0.67 (0.51, 0.88)	0.004
>120	0.96 (0.85, 1.09)	0.554
*P* for log-likelihood ratio test	0.039

*Adjusted: age, gender, BMI, smoking, hypertension, diabetes, Marfan syndrome, Bicuspid aortic valve, CRI, stroke, atherosclerosis, time to presentation, aortic diameter, aortic regurgitation, abdominal vessel involvement, arch vessel involvement, EF value, symptom, Cr, TG, TC, HDL, LDL, and management. BMI, body mass index; SBP, systolic blood pressure; Cr, creatinine; TG, triglyceride; TC, total cholesterol; HDL, high-density lipoprotein; LDL, low-density lipoprotein; CRI, chronic renal insufficiency; EF, ejection fraction*.

### Subgroup Analysis

We used gender, age, smoking, BMI, hypertension, diabetes, Marfan syndrome, bicuspid aortic valve, CRI, stroke, atherosclerosis, time to presentation, aortic diameter, aortic regurgitation, abdominal vessel involvement, arch vessel involvement, EF value, symptom, and management as the stratification variables to detect the development of effect sizes in these variables (Table 5). We observed that the deviation in the male population is more pronounced (*p* for interaction = 0.036, 0.88 with male vs. 1.00 with female). A similar trend was observed in non-diabetics (*p* for interaction = 0.020, 0.90 with non-diabetics vs. 1.37 with diabetics).

**Table 5:** Results of subgroup analysis and interaction analysis (SBP per 10 increments).

**Characteristic**	**No**.	**OR**	**95%CI Low**	**95%CI High**	***P* (interaction)**
Age (years)					0.682
<60	536	0.90	0.85	0.96	
≥60	167	0.93	0.83	1.03	
Gender					0.036*
Male	536	0.88	0.83	0.93	
Female	167	1.00	0.90	1.11	
BMI (Kg/m^2^)					0.920
<18.5	22	0.98	0.68	1.41	
≥18.5, <23	199	0.91	0.82	1.01	
≥23	482	0.90	0.85	0.96	
Smoking					0.483
no	506	0.92	0.86	0.98	
yes	197	0.88	0.79	0.98	
Hypertension					0.186
no	225	0.95	0.85	1.05	
yes	478	0.87	0.82	0.93	
Diabetes					0.020*
no	681	0.90	0.85	0.95	
yes	22	1.37	0.94	2.01	
Marfan syndrome					0.059
no	683	0.90	0.86	0.95	
yes	20	1.34	0.87	2.08	
Bicuspid aortic valve					0.928
no	695	0.91	0.86	0.96	
yes	8	0.85	0.47	1.55	
CRI					0.273
no	685	0.91	0.86	0.96	
yes	18	0.76	0.55	1.06	
Stroke					0.679
no	679	0.90	0.86	0.95	
yes	24	0.96	0.74	1.23	
Atherosclerosis					0.142
no	655	0.92	0.87	0.97	
yes	48	0.77	0.61	0.98	
Time to presentation (h)					0.656
Low (1.00**–**14.00)	256	0.90	0.84	0.97	
Middle (15.00**–**58.00)	254	0.86	0.78	0.95	
High (59.00**–**288.00)	193	0.92	0.78	1.09	
Aortic diameter (mm)					0.227
<35	67	0.82	0.67	1.00	
≥35	495	0.93	0.87	0.99	
Aortic regurgitation					0.155
no	389	0.88	0.82	0.94	
yes	314	0.95	0.88	1.03	
Abdominal vessel involvement					0.870
no	520	0.91	0.86	0.97	
yes	183	0.90	0.81	1.01	
Arch vessel involvement					0.757
no	482	0.91	0.86	0.97	
yes	221	0.90	0.82	0.99	
EF value (%)					0.551
<50	25	0.83	0.59	1.17	
≥50	528	0.92	0.86	0.98	
Symptom					0.115
Chest pain	581	0.91	0.86	0.96	
Back pain	22	0.67	0.45	0.99	
Abdominal pain	25	0.95	0.71	1.27	
Syncope	18	0.55	0.29	1.06	
Management					0.302
Medical	219	0.95	0.84	1.07	
Endovascular	43	1.08	0.71	1.63	
Surgical	441	0.85	0.76	0.95	

*BMI, body mass index; CRI, chronic renal insufficiency; EF, ejection fraction*.

## Discussion

In the fully adjusted model, we found that admission SBP negatively correlated with in-hospital mortality after fine-tuning other covariates. The model-based effect sizes can be interpreted as that a 10 mmHg rise in admission SBP is associated with 12% lower odds of in-hospital mortality. We also found non-linearity between admission SBP and in-hospital mortality. On the left side of the inflection point (SBP ≤ 120 mmHg), risk of in-hospital deaths in AAD patients was condensed by 33% for each extra 10 mmHg of admission SBP. On the right side of the inflection point (SBP > 120 mmHg), this relationship could not be observed [0.96 (95%CI 0.85–1.09), *p* = 0.554]. Moreover, subgroup analysis identified a stronger association between admission SBP and in-hospital mortality in males and non-diabetics.

In hypertension patients, lowering blood pressure decreases risk of cardiovascular events and death (10, 11), but the best target blood pressure is uncertain (12, 13). Large, randomized trials did not find benefit when blood pressure targets were <140/90 mmHg (14, 15). Additionally, several *post-hoc* analyses have demonstrated that the advantage of lowering blood pressure treatment might even be reversed below a certain threshold, which is called non-linear or J-curve phenomenon (16, 17). A series of studies have associated blood pressure with in-hospital mortality of AAD patients (18, 19). However, they did not perform non-linearity and subgroup analyses. Although Bossone et al. (20) found the relationship between in-hospital mortality and SBP in AAD patients to be non-linear, they did not elaborate on this. Consequently, the impact of this study was the innovation of a J-shaped curve and threshold effect on the link between admission SBP and in-hospital deaths in AAD patients.

Subgroup analyses are very important for scientific studies (21). They help us to a better understanding of the independent relationship between the admission of in-hospital deaths and SBP for AAD patients. In this study, we used gender, age, smoking, hypertension, BMI, diabetes, bicuspid aortic valve, Marfan syndrome, CRI, stroke, atherosclerosis, time to presentation, aortic diameter, aortic regurgitation, abdominal vessel involvement, arch vessel involvement, EF value, symptom, and management as stratification variables, of which interactions were observed in male and non-diabetes patients.

The following are the clinical values of this experiment: (1) to the best of our knowledge, this is the first report of the threshold effect between SBP admission and in-hospital death in AAD patients, (2) our findings may guide future studies on models (diagnostic and predictive) of in-hospital death rates in AAD patients. However, the improvement of research results is needed for clinical treatment decisions.

There are certain strengths in this study. (1) Non-linearity is higher, offering room for exploration; (2) As a result of observational study, strict statistical adjustments were used to reduce residual confounders; (3) Target independent variables were handled equally as continuous and categorical variables. Such an approach can decrease contingency in data analysis and heighten the strength of results; (4) the effect modifier factor analysis improves data use and produces stable conclusions in diverse subgroups in this study.

This study has some limitations. First, our discoveries are based on a Chinese population, reducing the generalizability of our findings. Second, patients with little or high SBP may have died before getting to hospital, lowering the prevalence of sicker patients. Third, blood pressure pharmacologic management in AAD patients was done by physicians in accordance with recent guidelines but this was not protocol-driven. Our current data cannot prove whether this factor will affect patient outcomes. Lastly, pre-hospital medication and emotion were not available, which may affect blood pressure.

## Conclusions

The relationship between admission SBP and in-hospital mortality is not linear. At ≤ 120 mmHg, SBP correlation to in-hospital mortality is negative.

## Data Availability Statement

The datasets generated for this study are available on request to the corresponding author.

## Ethics Statement

The studies involving human participants were reviewed and approved by the Second Xiangyang Hospital of Central South University. Written informed consent for participation was not required for this study in accordance with the national legislation and the institutional requirements.

## Author Contributions

The manuscript writing and collection of patient information was done by GY. Data collection was done by WP, HH, XL, YZ, and XP. The patients' general indices were analyzed and interpreted by XC. The final manuscript was read and approved by all authors.

## Conflict of Interest

The authors declare that the research was conducted in the absence of any commercial or financial relationships that could be construed as a potential conflict of interest.
