# Supplementary material for: Admission Systolic Blood Pressure and In-hospital Mortality in Acute Type A Aortic Dissection: A Retrospective Observational Study
Source: Front Med (Lausanne). 2021 Jul 20;8:542212. doi: 10.3389/fmed.2021.542212 (PMC8329236; doi:10.3389/fmed.2021.542212)
Supplement: Supplementary file 1 [file Table_1.DOCX]

**S-table 1.The causes of death in each quartile of operation patients.**

| Characteristic | Systolic blood pressure (mmHg) (Quarter) | | | | P-value |
| --- | --- | --- | --- | --- | --- |
|  | Q1(64-125) | Q2(126-144) | Q3(145-164) | Q4(165-233) |  |
| Causes of death |  |  |  |  | 0.999 |
| stroke | 9 (42.86%) | 5 (41.67%) | 3 (37.50%) | 4 (44.44%) |  |
| LCOS | 3 (14.29%) | 3 (25.00%) | 1 (12.50%) | 1 (11.11%) |  |
| MODS | 3 (14.29%) | 2 (16.67%) | 2 (25.00%) | 2 (22.22%) |  |
| Cardiac tamponate | 2 ( 9.52%) | 1 ( 8.33%) | 1 (12.50%) | 1 (11.11%) |  |
| Sepsis shock | 4 (19.05%) | 1 ( 8.33%) | 1 (12.50%) | 1 (11.11%) |  |

Abbreviations: LCOS, Low cardiac output syndrome; MODS, Multiple Organ Dysfunction Syndrome.

**S-table 2.The rates of in-hospital complications in each quartile of medical management patients.**

| Characteristic | Systolic blood pressure (mmHg) (Quarter) | | | | P-value |
| --- | --- | --- | --- | --- | --- |
|  | Q1(64-125) | Q2(126-144) | Q3(145-164) | Q4(165-233) |  |
| In-hospital complications |  |  |  |  | 0.985 |
| Stroke | 8 (11.0%) | 4 ( 8.9%) | 5 (10.2%) | 4 ( 8.7%) |  |
| Coma | 5 ( 6.8%) | 2 ( 4.4%) | 2 ( 4.1%) | 2 ( 4.3%) |  |
| Myocardial ischemia/infarction | 8 (11.0%) | 7 (15.6%) | 5 (10.2%) | 2 ( 4.3%) |  |
| Mesenteric ischemia/infarction | 2 ( 2.7%) | 1 ( 2.2%) | 1 ( 2.0%) | 3 ( 6.5%) |  |
| Acute renal failure | 15 (20.5%) | 9 (20.0%) | 8 (16.3%) | 10 (21.7%) |  |
| Cardiac tamponade | 26 (35.6%) | 14 (31.1%) | 19 (38.8%) | 16 (34.8%) |  |
